# Flash Memory Featuring Low-Voltage Operation by Crystalline ZrTiO_4_ Charge-Trapping Layer

**DOI:** 10.1038/srep43659

**Published:** 2017-03-08

**Authors:** Yung-Shao Shen, Kuen-Yi Chen, Po-Chun Chen, Teng-Chuan Chen, Yung-Hsien Wu

**Affiliations:** 1 Department of Engineering and System Science, National Tsing Hua University, Hsinchu 30013, Taiwan

## Abstract

Crystalline ZrTiO_4_ (ZTO) in orthorhombic phase with different plasma treatments was explored as the charge-trapping layer for low-voltage operation flash memory. For ZTO without any plasma treatment, even with a high k value of 45.2, it almost cannot store charges due the oxygen vacancies-induced shallow-level traps that make charges easy to tunnel back to Si substrate. With CF_4_ plasma treatment, charge storage is still not improved even though incorporated F atoms could introduce additional traps since the F atoms disappear during the subsequent thermal annealing. On the contrary, nevertheless the k value degrades to 40.8, N_2_O plasma-treated ZTO shows promising performance in terms of 5-V hysteresis memory window by ±7-V sweeping voltage, 2.8-V flatband voltage shift by programming at +7 V for 100 μs, negligible memory window degradation with 10^5^ program/erase cycles and 81.8% charge retention after 10^4^ sec at 125 °C. These desirable characteristics are ascribed not only to passivation of oxygen vacancies-related shallow-level traps but to introduction of a large amount of deep-level bulk charge traps which have been proven by confirming thermally excited process as the charge loss mechanism and identifying traps located at energy level beneath ZTO conduction band by 0.84 eV~1.03 eV.

NAND flash memory devices have become one of the fastest growing segments of semiconductor memories because smartphones, hand-held gadgets and embedded applications all favor flash memory as a lightweight, fast, small and reliable alternative to disk storage. Conventionally, poly-Si that is electrically conductive has been adopted as the charge storage media for NAND memory. However, the relatively thick tunnel oxide (6~7 nm) and inter-poly dielectric (10~13 nm) not only limit vertical down scaling, but lead to high program/erase voltage in the range of 17~19 V. Due to these limitations, poly-Si has been replaced with Si_3_N_4_ which possesses discrete charge-trapping sites and is fundamentally more scalable as one moves to subsequent generations of 3D NAND products, the most viable solutions for Tera-bit arrays. Therefore most NAND vendors adopt Si_3_N_4_ as the charge-trapping layer (CTL) for their 3D memory arrays. Unfortunately, Si_3_N_4_ is not a good CTL because of shallow-level traps, a small conduction band offset (ΔE_c_) of 1.1 eV with SiO_2_, a large ΔE_c_ of 2.1 eV with Si substrate and a relatively low dielectric constant (k) of 7. An ideal CTL should have deep-level traps as well as a larger ΔE_c_ with tunnel oxide for desirable charge storage and retention, a smaller ΔE_c_ with Si substrate for better charge injection, and a higher k value so that the electric field (E) over the SiO_2_ tunnel oxide can be higher due to electric flux density (D) continuity^1^, making higher program/erase speed and lower operation voltage. Many high-k dielectrics such as HfO_2_^2^, ZrO_2_^3^, La_2_O_3_^4^, and Y_2_O_3_^5^ have been extensively explored and exhibited promising memory characteristics. However, most high-k based CTLs are of amorphous phase with their k values rarely exceeding 25, limiting further scaling in operation voltage. Recently, phase transformation of a high-k dielectric from amorphous phase to crystalline one has attracted considerable interest since it provides an effective method to enhance the k value without compromising the bandgap^6^,^7^,^8^,^9^,^10^,^11^,^12^. The widely developed crystalline CTL mainly focuses on ZrO_2_^6^,^7^,^8^,^9^ with a k value of 38.7 in tetragonal phase^6^ and 32.8 in cubic phase^8^. Employing a crystalline CTL indeed achieves lower voltage operation as compared to conventional amorphous CTL.

To achieve a larger memory window at lower operation voltage, crystalline ZrTiO_4_ (ZTO) in orthorhombic phase with a high k value of 45.2^13^ was adopted as the CTL in this work. Note that besides the higher k value, due to the incorporation of TiO_2_ which is a small-bandgap dielectric with near-zero ΔE_c_ with Si substrate^14^, ZTO has a band structure more favorable than typical ZrO_2_ or HfO_2_ for the application of CTL in terms of a large ΔE_c_ of 2.2 eV with tunnel SiO_2_ and a smaller ΔE_c_ of 1.0 eV with Si substrate^15^, as the comparison of band alignment among different dielectrics shown in Fig. 1. It is worth noting that orthorhombic ZTO has been verified as a viable gate dielectric for aggressively scaled MOSFETs^13^ and therefore is not a desirable candidate as the CTL due to less amount of charge-trapping sites. To circumvent this issue, N_2_O or CF_4_ plasma treatment on ZTO was carried out to introduce charge traps while preserving the high k value of orthorhombic ZTO. Furthermore, the impact of various treatments on memory device characteristics were also analyzed. The reason to adopt N_2_O or CF_4_ plasma treatment lies in the fact that incorporation of anions such as nitrogen^16^,^17^,^18^ or fluorine^19^,^20^ into the CTL has been proposed to improve the characteristics of memory devices by introducing more deep-level traps. The results indicate that memory devices based on ZTO without any plasma treatment reveal negligible memory characteristics. However, with additional N_2_O plasma treatment on ZTO, the memory performance significantly enhances which is evidenced by a large memory window of 5.0 V with ±7 V program/erase voltage, high operation speed of 2.8 V flatband voltage shift by programming at +7 V for 100 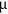
s, negligible memory window degradation up to 10^5^ operation cycles, and 18.2% charge loss after 10^4^-second operation at 125 °C. Most importantly, the process is fully compatible with existent ULSI technology and paves the way to adopt a crystalline ZTO as the charge-trapping layer for future next-generation low-power flash memory.

## Results and Discussion

**Figure 2:**
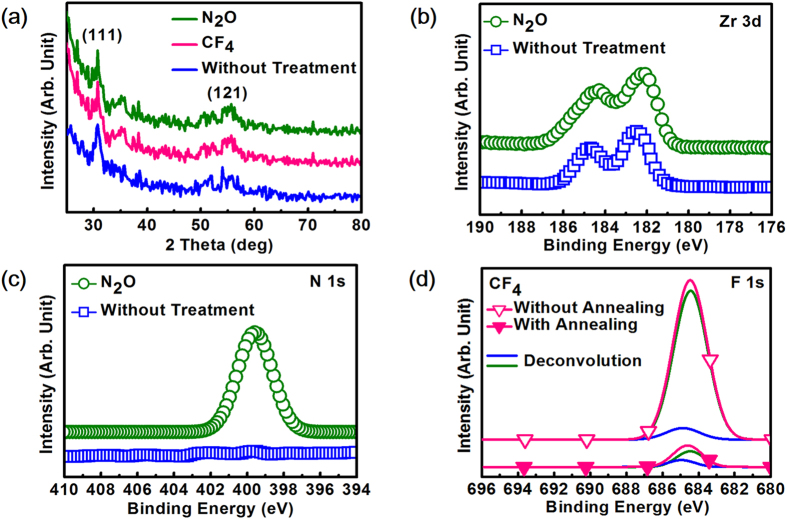
(**a**) XRD patterns for ZTO with different plasma treatments after annealing. **(b)** Zr 3d XPS spectra for annealed ZTO with and without N_2_O plasma treatment. **(c)** Impact of N_2_O plasma treatment on N 1s spectra for ZTO after annealing. **(d)** XPS F 1s spectra for CF_4_ plasma-treated ZTO with and without 700 °C thermal annealing. Deconvolution results for both conditions are also shown.

The x-ray diffraction pattern (XRD) patterns of the ZTO with different plasma treatments after thermal annealing are represented in Fig. 2a. As expected, the XRD pattern for untreated ZTO displays diffraction peaks at 30.7 ° and 55.6 °, implying the crystallization of the ZTO in orthorhombic phase with (111) and (121) orientation. For N_2_O and CF_4_ plasma-treated ZTO, nearly identical patterns to those of untreated ZTO are observed which suggest the formation of orthorhombic phase even though nitrogen or fluorine atoms are introduced. Note that without thermal annealing, ZTO is of amorphous phase no matter what plasma treatment is adopted which is proven by the lack of any diffraction peaks (not shown). In fact, as the annealing temperature is below 700 °C, the ZTO remains amorphous since no discernable peaks can be observed which means that 700 °C is the minimum thermal budget to crystallize ZTO from amorphous to orthorhombic phase. The temperature-dependent crystallization behavior in this work is consistent with others reported in the literature^21^, although some researches have shown crystallization temperature below 700 °C^22^. It is worth mentioning that TiO_2_ and ZrO_2_ can be respectively crystallized in anatase or monoclinic phase below 600 °C^23^. However, no phase separation (formation of either TiO_2_ or ZrO_2_) is observed after the annealing in this work which is desirable because phase separation would result in the need for significantly higher temperatures in order to obtain crystalline ZTO upon thermal treatment. The phenomenon that no phase separation suggests that the process to deposit ZTO in this work allows the mixing of individual components on an atomic scale, which in turn causes a lower temperature to crystalize the ZTO film. It is worthy to be mentioned that compared to untreated ZTO, the (111) XRD peak of N_2_O plasma-treated ZTO shifts to lower diffraction angle by 0.15°. Typically, XRD peak shift toward smaller angle has been reported in the literature when the cell volume increases^24^ and it well explains the peak shift in this work since incorporating nitrogen ions (ionic radius of 171 pm) to replace with lattice oxygen ions (ionic radius of 140 pm) in ZTO will cause increase in cell volume, implying N_2_O plasma treatment indeed introduces nitrogen into ZTO. To investigate the chemical states of nitrogen and fluorine in ZTO, x-ray photoelectron spectroscopy (XPS) measurement was carried out on different samples and Fig. 2b displays the impact of N_2_O plasma treatment on Zr 3d spectra for ZTO after annealing. For untreated ZTO, doublet peaks at binding energy (BE) of 182.5 eV and 184.7 eV respectively correspond to the Zr 3d_5/2_ and Zr 3d_3/2_ signals. The respective BE peak is quite close to that reported in the literature^25^ and the Zr/Ti ratio of 1.2 is estimated from the composition analysis, approaching stoichiometric ZrTiO_4_ where the ratio is 1.0. For N_2_O plasma-treated ZTO, the peaks shift by approximately 0.2 eV-0.3 eV toward lower BE as compared to untreated ZTO. This BE shift can be understood by the fact that some nitrogen atoms have been incorporated into ZTO to substitute for the lattice oxygen atoms. Because the electronegativity of N (3.0) is smaller than that of O (3.5), the substitution effect would make electrons transfer from nitrogen atoms to oxygen atoms, lowering the BE of the Zr 3d core electrons. The inference that nitrogen atoms are incorporated into ZTO can be further evidenced by the N 1s spectra for ZTO with and without N_2_O plasma treatment shown in Fig. 2c. A peak at BE of 399.7 eV is observed for N_2_O plasma-treated ZTO while no such peak can be found for ZTO without any treatment, suggesting the incorporation of nitrogen atoms into ZTO by N_2_O plasma treatment. Note that the N 1 s BE peak of 399.7 eV is higher than that of stoichiometric TiN and ZrN which respectively correspond to 396.7 eV and 397.1 eV, indicating that nitrogen atoms strongly react with oxygen atoms and Ti-O-N or Zr-O-N bonds are very likely to form after N_2_O plasma treatment. For the behavior of CF_4_-treated ZTO, the impact of thermal annealing on F 1 s spectra is shown in Fig. 2d. Also shown in the figure are the deconvolution results for both conditions. Without annealing, F1s XPS signal for CF_4_-plasma treated ZTO is composed of a major and minor signal with peak at 684.4 eV and 685.0 eV by deconvolution which respectively correspond to Ti-F and Zr-F bond according the literature^26^,^27^. That is, for CF_4_-plasma treated ZTO, most F atoms are incorporated into the dielectric in the form of Ti-F bonds. After annealing at 700 °C, however, the intensity of Ti-F bonds drastically decreases while that of Zr-F bonds only slightly changes. The results indicate that the significantly reduced F1s XPS signal after annealing is mainly caused by the intensity attenuation of Ti-F bonds rather than Zr-F bonds. The most likely reason of the phenomenon can be explained by the fact that the thermal energy at the annealing temperature is high enough to break Ti-F bonds, creating mobile F ions and out diffusing from the ZTO. The inference can be proven by investigating the bond dissociation energy (enthalpy) which is also referred to as bond disruption energy, or bond strength^28^. For Ti-F, Zr-F and Si-F bonds, the bond dissociation energy are found to be 569.0 eV, 627.2 eV and 576.4 eV respectively. Since it has been demonstrated that Si-F bond can be broken (dissociated) at 600 °C for F-incorporated SiO_2_^29^,^30^,^31^ and the required energy to dissociate Si-F bonds is higher than that of Ti-F, it can be inferred that Ti-F bonds can dissociated at 700 °C annealing temperature. On the other hand, because the bond dissociation energy for Zr-F is higher than Ti-F, it is possible for these bonds to remain unbroken at the annealing temperature. From the concept of bond dissociation energy, it well explains why the signal intensity of Ti-F attenuates significantly while that of Zr-F only changes slightly.

**Figure 3:**
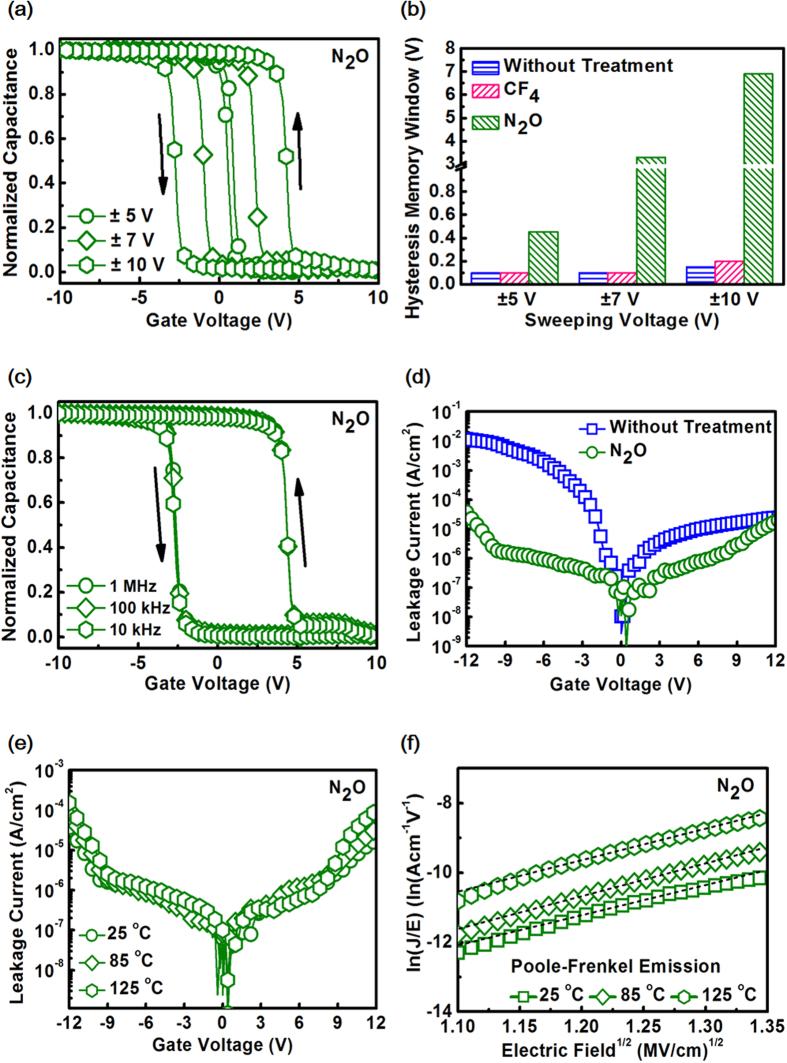
Characteristics of memory window and leakage current for memory devices with SiO_2_ as the blocking oxide. **(a)** Bi-directional C-V hysteresis for devices with N_2_O plasma-treated ZTO as the CTL. **(b)** Dependence of sweeping voltage on hysteresis memory window for CTL with various treatments. **(c)** C-V curves for for devices with N_2_O plasma-treated ZTO measured in the the frequency range of 10 kHz to 1000 kHz. **(d)** Leakage current comparison for N_2_O plasma-treated ZTO and untreated ZTO. **(e)** Temperature-dependent leakage current for N_2_O plasma-treated ZTO measured at 25 °C, 85 °C and 125 °C. **(f)** P-F emission at 25 °C, 85 °C and 125 °C for devices with N_2_O plasma-treated ZTO as the CTL.

Shown in Fig. 3 are the characteristics of memory window and leakage current for memory devices with SiO_2_ as the blocking oxide. Figure 3a shows the bi-directional capacitance-voltage (C-V) hysteresis curves for memory devices with N_2_O plasma-treated ZTO as the CTL by applying different sweeping voltages measured at 1 MHz. As the sweeping voltage increases from ±5 V, ±7 V and ±10 V, the hysteresis memory window (flatband voltage shift, ΔV_FB_) increases from 0.5 V, 3.3 V, and 6.9 V. The counterclockwise hysteresis is attributed to the injection of deep-inversion electrons from the substrate^32^. Figure 3b summarizes the dependence of sweeping voltage on hysteresis memory window for ZTO with various treatments. For untreated ZTO, it shows negligible memory window and it can be inferred that a lot of oxygen vacancies arising from grain boundaries of ZTO contribute to shallow-level traps that can hardly store charges. For those with CF_4_ plasma treatment, the memory window is still too small. In fact, incorporation of F atoms into CTL has been verified as a viable avenue to enhance memory performance^19^,^20^ since it would be an excellent passivant to remove shallow-level defects (traps) which are not effective charge storage media and to strengthen the dielectric films due to its strong electronegativity. Furthermore, fluorine incorporation has also shown the capability to induce deep-level traps. Unfortunately, the advantages of employing fluorine treatment on CTL is not observed in this work and this phenomenon can be explained by the XPS analysis shown in Fig. 2d from which the content of fluorine atoms in the ZTO greatly decreases to be less than 1% after annealing and it is the out diffusion of fluorine atoms that makes the memory characteristics comparable to those of the memory with untreated ZTO. The reason why N_2_O plasma induces desirable memory window can be understood as follows. In the environment of plasma, N_2_O will be decomposed into O and NO radicals. O radicals help passivate oxygen vacancies stemming from grain boundaries and therefore shallow-level traps can be mitigated. In addition, interface traps between CTL and tunnel SiO_2_ can also be suppressed. Furthermore, NO radicals are responsible for generation of deep-level bulk charge traps which are favorable for charge storage without tunneling back to substrate or through blocking oxide. In addition, form the capacitance value, the k value for untreated ZTO and N_2_O plasma-treated ZTO are respectively 45.2 and 40.8. Although the k value degrades due to nitrogen incorporation which is consistent with ZrO_2_^8^ and TiO_2_^33^, the k value of N_2_O plasma-treated ZTO is still higher than most CTLs reported in the literature and holds the advantages of reducing operation voltage by enhancing the electric field over tunnel SiO_2_^1^. Since only memory devices with N_2_O plasma-treated ZTO show appreciable memory window, the following discussion focus on the split condition. To ascertain that the charge storage effect is primarily resulted from the bulk traps in N_2_O plasma-treated ZTO rather than from interface trap’s which would lead to frequency dispersion and stretch out in C-V characteristics because interface traps generally give rise to time- or frequency-dependent C-V characteristics^34^,^35^, bi-directional C-V measurement in the range of 10 kHz to 1000 kHz was carried out and the results are shown in Fig. 3c. The frequency-independent C-V characteristics and memory windows prove that the charge storage media are not interface traps but bulk traps. Besides the significant memory window, the greatly enhanced charge storage capability for N_2_O plasma-treated ZTO is also evidenced by the tunneling current characteristics versus electric field over CTL as shown in Fig. 3d. It is clearly that the current for N_2_O plasma-treated ZTO drastically decreases as compared to that of untreated ZTO and the tunneling current reduction can be ascribed to the fact that more injected electrons from the substrate are captured in the bulk traps. Figure 3e reveals the leakage current for N_2_O plasma-treated ZTO measured at 25 °C, 85 °C and 125 °C. The strong temperature dependence of the leakage current suggests a possible Poole-Frenkel emission (P-F) or a Schottky emission (SE) conduction mechanism. As shown in Fig. 3f from which P-F relationship well fits the experimental data well, it confirms that the current conduction mechanism is dominated by P-F emission, implying a large amount of deep-level traps existence in the N_2_O plasma-treated ZTO. The result is reasonable because the transport of charge for P-F emission is mainly between localized electronic states in the energy gap caused by structural defects and N_2_O plasma treatment indeed generate such defects in ZTO.

**Figure 4:**
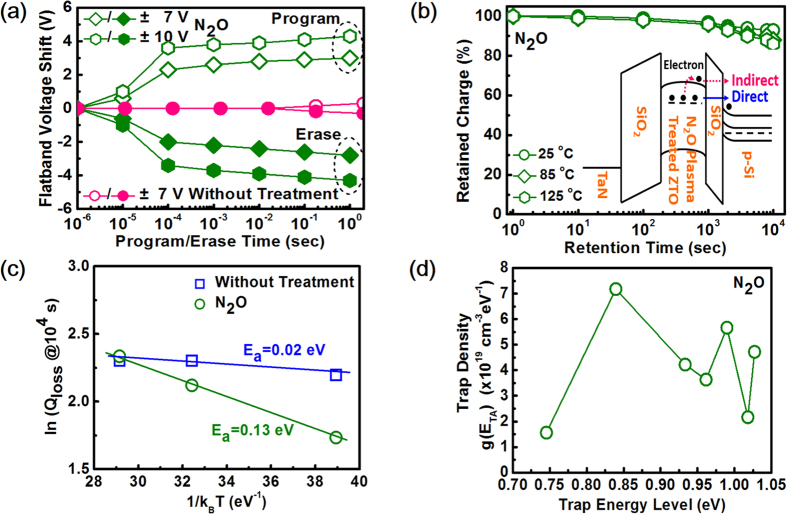
Operation speed and retention performance for memory devices with SiO_2_ as the blocking oxide. **(a)** P/E transient characteristics for devices with N_2_O plasma-treated ZTO as the CTL. The same characterisitcs for those with untreated ZTO are also shown for comparison. **(b)** Data retention for devices with N_2_O plasma-treated ZTO as the CTL measured at various temperatures. The inset shows the energy band daigram under the retention mode to illustrate different charge loss mechanisms. **(c)** Arrhenius plot of the charge loss for untreated and N_2_O plasma-treated devices at 10^4^ s retention time in the range of 25 °C to 125 °C. **(d)** Extracted trap density as a function of the trap energy level for N_2_O plasma-treated ZTO based on Yang and White electron retention model.

Figure 4 shows the operation speed and retention performance for memory devices with SiO_2_ as the blocking oxide. The program/erase (P/E) transient characteristics for N_2_O plasma-treated ZTO are shown in Fig. 4a to evaluate its operation speed. The data for untreated ZTO are also shown for comparison. For untreated ZTO, because the traps in untreated ZTO are of shallow-level ones that can hardly store charges, untreated ZTO shows negligible ΔV_FB_ even the program/erase time prolongs to 1 sec. For N_2_O plasma-treated ZTO, a 2.0-V ΔV_FB_ was achieved by programming at +7 V for 100 μs. With the same program time, ΔV_FB_ increases to 3.4 V as the program voltage increases to +10 V. Similar memory performance can also be found for erase operation with opposite voltage polarity. The operation voltage less than 10 V with a relatively large memory window is desirable for low-power green electronics since currently flash memory devices are operated in the range of 15–17 V. This low operation voltage is not only due to the merits of N_2_O plasma-treated ZTO which corresponds to a high k value of 40.8, but also more charge-trapping sites with deeper energy level introduced by N_2_O plasma treatment. Note that typically the trap level correlates to retention performance rather than program voltage. However, if the trap level is too shallow, charges are easily to be de-trapped and consequently leads to degradation of charge trapping efficiency. If the phenomenon occurs, to achieve a specific ΔV_FB_, a higher program voltage is required to inject more charges to compensate for the de-trapped charges. On the contrary, if a lot of traps with relatively deep level can be formed in the charge trapping layer, the charge trapping efficiency can be enhanced under a certain amount of charge injection. In other words, a lower voltage for charge injection is required to achieve the specific ΔV_FB_. To better understand the mechanism behind the charge loss, the trap activation energy is determined from the temperature-dependent charge retention behavior^36^,^37^,^38^ measured from 25 °C (298 K) to 125 °C (473 K) which is shown in Fig. 4b. For N_2_O plasma-treated ZTO, charge loss of 15.8% after 10^4^ sec at 125 °C is obtained while the charge loss reaches 96.8% for untreated ZTO under the same condition (not shown). By using the following expression:


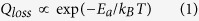


where *Q*_*loss*_ is the charge loss at 10^4^ s, *E*_*a*_ is the activation energy for the charge loss, *k*_*B*_ is the Boltzmann constant, and 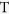 is absolute temperature, *E*_*a*_ can be extracted from the Arrhenius plot of log (charge loss) vs. 1/*k*_*B*_*T* for devices based on untreated and N_2_O plasma-treated ZTO shown in Fig. 4c. The extracted *E*_*a*_ for untreated and N_2_O plasma-treated ZTO are 0.02 eV and 0.13 eV respectively. The much smaller *E*_*a*_ for untreated ZTO suggests that trap-to-band tunneling is the charge loss mechanism which is a direct charge loss process with temperature independent behavior as shown in the inset of Fig. 4b^36^. The significant charge loss for untreated ZTO implies that trap-to-band tunneling is mainly responsible for seriously deteriorated data retention. The pronounced trap-to-band tunneling may be caused by electrons trapped at the shallow levels which make lower tunneling barrier and/or electrons trapped near the ZTO/SiO_2_ which makes a shorter tunneling distance. On the contrary, the larger *E*_*a*_ for N_2_O plasma-treated ZTO indicates that the charge loss corresponds to indirect process from which electrons need to be thermally excited to conduction band of the CTL and then tunnel back to Si substrate, as illustrated in the inset of Fig. 4b. The relatively good charge retention for N_2_O plasma-treated ZTO suggests that the indirect process is insignificant and this phenomenon is primarily ascribed to the deep-level traps that make the stored electron less susceptible to external thermal energy in the temperature range. This result also infers that N_2_O plasma treatment indeed introduces traps with deeper level as compared with untreated ZTO. Considering the thermal activation as the dominant charge loss mechanism for devices based on N_2_O plasma-treated ZTO, the trap energy level can be further extracted by Yang and White electron retention model^39^,^40^.


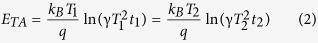



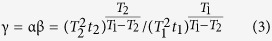



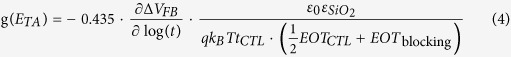


where E_TA_ is the energy level from the conduction band edge of the CTL, γ is a whole coefficient composed of two parameters of α and β where α is a combination of the temperature independent constants and β is a coefficient of tunneling probability through the tunneling oxide, T_1_ and T_2_ represent the retention characteristics of the sample at two temperatures after programming all the traps with electrons, t_1_ and t_2_ are the times when the two 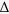
V_FB_ have the same value, EOT is the equivalent oxide thickness, and g(E_TA_) represents the simulated dielectric trap density derived by Yang and White electron retention model. Using equation (1), (2), and (3), the trap density distribution as a function of trap level energy can be plotted in Fig. 4d and the trap density distribution can be observed between 0.84 eV~1.03 eV beneath the ZTO conduction band and this trap energy level is deeper than Si_3_N_4_ CTL reported by other’s group^41^, another evidence that N_2_O plasma treatment indeed induces deep-level traps.

As mentioned previously, crystalline dielectric has been adopted as the charge trapping layer^6^,^7^,^8^,^9^,^10^,^11^,^12^ and even blocking oxide^10^,^42^. Since the crystalline dielectrics used in the devices are of poly-crystalline phase, one concern may be the variation of number of grains in a cell which would lead to undesirable non-uniformity of device performance. In fact, the concern has been verified by evaluating the dependence of channel area on device performance for poly-Si thin film transistors (TFTs) where the channel is composed of many poly-Si grains^43^. From the results, it is observed that as the channel area becomes smaller, the uniformity of device performance gets better in terms of tighter distribution. It can be explained by the fact that when a poly-Si TFT scales, the channel area becomes almost the same as the grain size of poly-Si. Therefore, TFTs are probably formed on the single crystalline grain and the dependence of number of grains on performance is alleviated for aggressively scaled poly-Si TFTs. Based on the phenomenon, it suggests that when the flash memory cells continue to scale, it is likely to be smaller than the grain size of the crystalline dielectric. In other words, for an aggressively scaled memory cell, it is possible to have only one grain in the charge trapping layer and therefore the uniformity of device performance would not be degraded as the cell size becomes smaller. In this work, the grain size for annealed ZTO is in the range of 33~46 nm which is extracted from x-ray diffraction pattern by Scherrer equation. With additional N_2_O plasma treatment, the grain size is almost in the same range. Based on the extracted grain size, the charge trapping layer may contain only one grain when the memory cell is smaller than 33 nm. For advanced 3D flash, the cell size is much smaller the grain size and thus the uniformity issue will become less pronounced. On the other hand, from 2015 International Technology Roadmap for Semiconductors (ITRS)^44^, the prototypical and emerging nonvolatile memory devices include phase change memory (PCM) and ferroelectric memory (ferroelectric FET and ferroelectric tunnel junction) where poly-crystalline dielectrics are respectively used for phase change and dipole orientation change. Therefore, employing crystalline dielectrics for memory devices is one of the future trends and variation in grain numbers for the crystalline dielectrics is not an issue for scaled devices.

**Figure 5:**
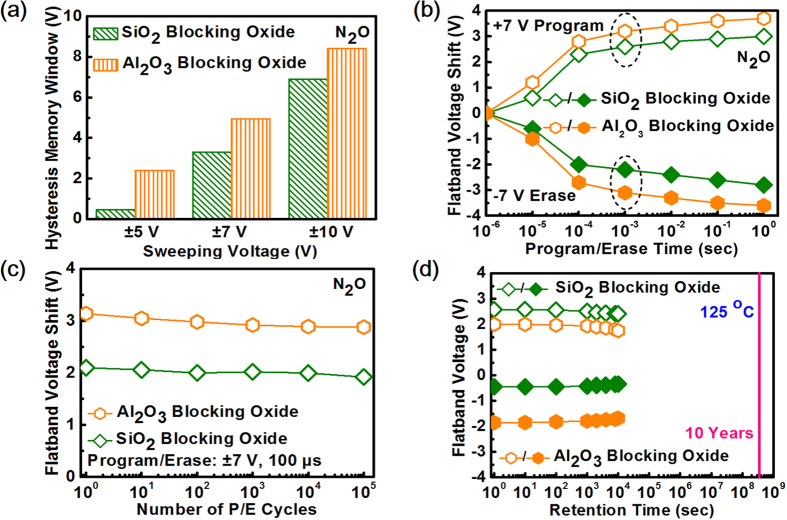
Impact of blocking oxides on memory characteristics for N_2_O plasma-treated ZTO. **(a)** Dependence of sweeping voltages on hysteresis memory window for devices based on N_2_O plasma-treated ZTO with different blocking oxides. **(b)** P/E transient characteristics for devices with different blocking oxides under ±7 V operation voltage. **(c)** Endurance characteristics for devices with different blocking oxides by applying ±7 V with 100-μs gate pulse width. **(d)** Retention characteristics at 125 °C for devices with different blocking oxides.

Having confirmed N_2_O plasma-treated ZTO as a viable CTL, SiO_2_ blocking oxide was replaced with Al_2_O_3_ and the hysteresis window are enhanced under various sweeping voltages as shown in Fig. 5a. The memory window increases from 3.3 V to 5.0 V by changing the blocking oxide from SiO_2_ to Al_2_O_3_ under ±7 V sweeping voltage, proving the effectiveness to enhance the E field over tunnel SiO_2_. The desirable property is also manifested in the P/E transient characteristics shown in Fig. 5b. With +7 V program voltage, ΔV_FB_ of 2.8 V can be obtained in 100 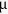
s for Al_2_O_3_ blocking oxide, which is only 2.2 V for SiO_2_ blocking oxide. Figure 5c shows the endurance characteristics for devices based on N_2_O plasma-treated ZTO using different blocking oxides. After P/E stressing for 10^5^ cycle, negligible ΔV_FB_ is achieved for both types of blocking oxides. Such a good endurance property is mainly due to the robust interface quality between ZTO/SiO_2_ and SiO_2_/Si which lead to few defects generated even after 10^5^-cycle P/E stressing. Figure 5d shows the normalized retained charges as a function of time measured at 125 °C for devices based on N_2_O plasma-treated ZTO with different blocking oxides. About 84.2% and 81.8% retained charge after 10^4^ sec at 125 °C are observed for the N_2_O plasma-treated devices with SiO_2_ and Al_2_O_3_ as the blocking oxide, respectively. The inferior retention for devices with Al_2_O_3_ as the blocking oxide may be due to the lower conduction band offset by 0.4 eV which is unfavorable for electron confinement. Nevertheless, the retention performance for those with Al_2_O_3_ is good enough for practical applications.

## Conclusion

High-k orthorhombic ZTO with various plasma treatments was explored as the charge-trapping layer for flash memory. Without any plasma treatment, ZTO can hardly store charges due to the shallow-level traps that make trapped electrons easy to tunnel back to Si substrate as evidenced by verifying trap-to-band tunneling as the charge loss mechanism. CF_4_ plasma-treated ZTO neither demonstrates the discernible capability to store charges since F content greatly diminishes after thermal annealing, making F-induced traps disappear in the ZTO. In contrast, N_2_O plasma-treated ZTO exhibits promising memory characteristics with low voltage operation of 7 V in terms of a large hysteresis memory window of 5 V, flatband voltage shift of 2.8 V by programming for 100 μs, negligible memory window degradation up to 10^5^ program/erase cycles. Furthermore, 81.8% charges are retained after 10^4^ sec at 125 °C with thermally excited process confirmed as the charge loss mechanism. These desirable properties can be explained by the fact that N_2_O plasma treatment on ZTO not only passivates oxygen vacancies or/and shallow-level traps but introduces a large amount of deep-level bulk charge traps. It renders N_2_O plasma-treated ZTO a promising CTL for flash memory with low voltage operation.

## Methods

Standard RCA and HF-last cleaned p-type Si substrates with 1–10 Ω-cm resistivity were used as the starting materials. 3-nm SiO_2_ thermally grown on Si substrate by furnace was used as the tunnel oxide of the flash memory. Amorphous ZTO of 10.0 nm was deposited by an electron beam evaporation as the CTL. Then N_2_O (200 sccm) or CF_4_/O_2_ (200 sccm/20 sccm) plasma treatment was performed on the CTL at 300 °C for 60 sec for some samples to investigate how incorporation of nitrogen or fluorine atoms affects memory characteristics. Note that for CF_4_ plasma treatment, the low-concentration O_2_ was introduced to suppress the hydrogen and carbon in the plasma. Next, 10-nm SiO_2_ grown by plasma-enhanced chemical vapor deposition (PECVD) was deposited as the blocking oxide. In addition, 10-nm Al_2_O_3_, a dielectric with a higher k value than SiO_2_, was adopted as the blocking oxide for some samples by atomic layer deposition (ALD) with Trimethyl Aluminum (TMA) and H_2_O as the precursors to explore the impact of k value of blocking oxide on memory performance. Then TaN was subsequently deposited and patterned as the electrode with the area of 250 μm × 250 μm. Finally, all samples were annealed at 700 °C in N_2_ ambient for 30 sec to crystallize the CTL.

### Characterization

Electrical characterization for memory devices were performed by capacitance-voltage and current-voltage measurement, respectively by Keithley 4200-SCS semiconductor parameter analyzer and Agilent 4284 LCR meter with typical frequency of 10 kHz-1000 kHz and a 100 mV AC probing signal. In addition to electrical characterization, x-ray diffraction analysis with Cu Kα radiation (wavelength of 0.154056 nm) was adopted to confirm the crystallinity of the ZTO with various plasma treatments. Furthermore, x-ray photoelectron spectroscopy analysis was performed to investigate the bond structure of the ZTO film with different plasma treatments. In order to mitigate the surface charging effect on the insulating film, a charge neutralizer (low-energy electron gun) was employed for charge compensation in the XPS measurement.

## Additional Information

**How to cite this article:** Shen, Y.-S. *et al*. Flash Memory Featuring Low-Voltage Operation by Crystalline ZrTiO_4_ Charge-Trapping Layer. *Sci. Rep.* **7**, 43659; doi: 10.1038/srep43659 (2017).

**Publisher's note:** Springer Nature remains neutral with regard to jurisdictional claims in published maps and institutional affiliations.

## Figures and Tables

**Figure 1:**
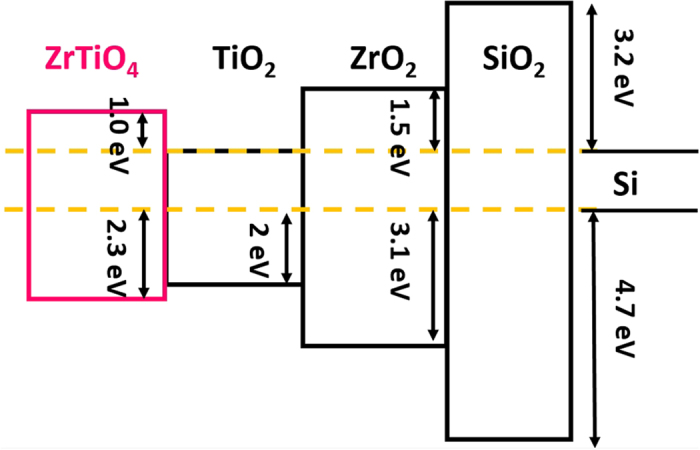
Band alignments for ZrTiO_4_, TiO_2_ and ZrO_2_ with respect to SiO_2_/Si system.
